# The “Reading the Mind in the Eyes” Test: Complete Absence of Typical Sex Difference in ~400 Men and Women with Autism

**DOI:** 10.1371/journal.pone.0136521

**Published:** 2015-08-27

**Authors:** Simon Baron-Cohen, Daniel C. Bowen, Rosemary J. Holt, Carrie Allison, Bonnie Auyeung, Michael V. Lombardo, Paula Smith, Meng-Chuan Lai

**Affiliations:** 1 Autism Research Centre, Department of Psychiatry, University of Cambridge, Cambridge, United Kingdom; 2 CLASS Clinic, Cambridgeshire and Peterborough NHS Foundation Trust, Cambridge, United Kingdom; 3 Department of Psychology, University of Edinburgh, Edinburgh, United Kingdom; 4 Department of Psychology and Center of Applied Neuroscience, University of Cyprus, Nicosia, Cyprus; 5 Centre for Addiction and Mental Health and Department of Psychiatry, University of Toronto, Toronto, Canada; 6 Department of Psychiatry, National Taiwan University Hospital and College of Medicine, Taipei, Taiwan; University of Tokyo, JAPAN

## Abstract

The “Reading the Mind in the Eyes” test (Eyes test) is an advanced test of theory of mind. Typical sex difference has been reported (i.e., female advantage). Individuals with autism show more difficulty than do typically developing individuals, yet it remains unclear how this is modulated by sex, as females with autism have been under-represented. Here in a large, non-male-biased sample we test for the effects of sex, diagnosis, and their interaction. The Eyes test (revised version) was administered online to 395 adults with autism (178 males, 217 females) and 320 control adults (152 males, 168 females). Two-way ANOVA showed a significant sex-by-diagnosis interaction in total correct score (F(1,711) = 5.090, *p* = 0.024, η_p_^2^ = 0.007) arising from a significant sex difference between control males and females (*p* < 0.001, Cohen’s *d* = 0.47), and an absence of a sex difference between males and females with autism (*p* = 0.907, *d* = 0.01); significant case-control differences were observed across sexes, with effect sizes of *d* = 0.35 in males and *d* = 0.69 in females. Group-difference patterns fit with the extreme-male-brain (EMB) theory predictions. Eyes test-Empathy Quotient and Eyes test-Autism Spectrum Quotient correlations were significant only in females with autism (*r* = 0.35, *r* = -0.32, respectively), but not in the other 3 groups. Support vector machine (SVM) classification based on response pattern across all 36 items classified autism diagnosis with a relatively higher accuracy for females (72.2%) than males (65.8%). Nevertheless, an SVM model trained within one sex generalized equally well when applied to the other sex. Performance on the Eyes test is a sex-independent phenotypic characteristic of adults with autism, reflecting sex-common social difficulties, and provides support for the EMB theory predictions for both males and females. Performance of females with autism differed from same-sex controls more than did that of males with autism. Females with autism also showed stronger coherence between self-reported dispositional traits and Eyes test performance than all other groups.

## Introduction

Autism spectrum conditions (henceforth “autism”) are diagnosed when an individual experiences social-communication difficulties, alongside unusually narrow interests and strong resistance to change, from early childhood and across their lifespan [1, 2]. The “mindblindness” theory [3] proposed that in autism a “theory of mind” (ToM, or “cognitive empathy”), sometimes referred to as “mind-reading” [4] or “mentalizing” [5], is impaired to varying degrees. ToM entails attribution and recognition of mental states in oneself or others [6] and to use such information to make inferences and predict behaviour. Toddlers with autism are impaired on tests of two precursors of ToM: joint attention and pretend play, both typically understood and produced by age 18 months [7–9]. Children and adults with autism are impaired in tasks assessing first-order false belief (i.e., recognizing that another person holds a belief that is not true), typically understood by 4 years old [10, 11]; second-order false belief (i.e., recognizing that another person holds a belief that another person believes something that is not true), typically understood by age 6 years old [12]; faux pas, typically understood by age 9 years old [13]; and reading subtle mental states from the eye region of the face [14], the voice [15], or in movie clips [16]. A ToM deficit is a parsimonious cognitive explanation for the social-communication difficulties that are ubiquitous in individuals with autism, across development, sex/gender (this is the preferred term for research in this field [17], but for simplicity “sex” is henceforth used in this article), IQ, or specific genetic syndromic forms [3, 11, 17, 18]. The consequence of this is difficulties in imagining the world from another’s perspective and tracking another’s mental states in real time during social interaction.

Individuals with autism also score significantly lower than typically developing individuals on the Empathy Quotient (EQ), a self-report [19, 20] or parent-report [21] measure of empathy. Empathy has at least two components [22]: “cognitive empathy” is synonymous with ToM, whereas “affective empathy” entails experiencing an appropriate emotion in response to another’s mental state (e.g., feeling pity in response to someone’s sadness, or feeling pleasure in response to someone’s happiness). Cognitive empathy is impaired in autism [23], whilst affective empathy likely remains intact [24, 25]. This profile in autism is the mirror image of those with psychopathic/antisocial personality, who often have intact or even superior cognitive empathy (e.g., they may analyse a victim’s mental states in great depth in order to identify their vulnerability) but reduced affective empathy [26–29]. For this reason, psychopaths may not care if their victim is in pain, and may even experience an inappropriate, self-centred emotion (such as pleasure) in response to someone else’s pain. Clinically, individuals with autism commonly report not *realising* that they have upset someone (a sign of impaired cognitive empathy), but feel remorse when it is pointed out to them that they have (a sign of intact affective empathy) [19, 30]. Unlike psychopaths, people with autism also commonly become upset if they hear someone else or an animal is suffering, and will stand up against injustice, quick to rush to the defence of the victim [26].

Self-report questionnaires (e.g., the EQ) reflect self-perception/evaluation, but not necessarily cognitive capability. The “Reading the Mind in the Eyes” Test (Eyes test) was therefore developed as a performance-based measure [14, 31]. It is an advanced ToM task involving mental state attribution and complex facial emotion recognition from photographs where only the eye region of the face is available. The Eyes test has been evaluated in over 250 studies to date, and has been found to have good reliability [32, 33]. Individuals with autism consistently perform less well relative to controls [16, 34–38]. The Eyes test also reveals a typical sex difference, with females scoring slightly but significantly higher than males [14, 39, 40].

Building on this typical sex differences, the finding that individuals with autism perform lower on the Eyes test than typically developing males fits a pattern predicted by the extreme-male-brain (EMB) theory of autism, which extends the Empathizing-Systemizing (E-S) theory of typical sex differences, and hypothesizes that at a cognitive level, characteristics of autism (in comparison to those of typically developing individuals) reflect an “extreme-male” form of specific characteristics typically showing sex difference in the general population [41]. In particular, the EMB theory predicts that on tests of empathy, the pattern of scores will be “typically developing females > typically developing males > people with autism”. The EMB theory also predicts that on measures of systemizing (the drive to analyse or construct a rule-based system, and to predict how a system works), the pattern of results will be the opposite. These patterns of results have been found using the EQ and the Systemizing Quotient (SQ) in both adults [20, 42] and children [21]. The idea that the autistic brain may be evolved to “hyper-systemize” [43] can explain the narrow interests shown by individuals with autism and their strong “obsessive insistence on the preservation of sameness” [44]. This is because—like science itself—systemizing involves holding all variables constant whilst varying just one variable at a time, in order to identify a law. Similarly, like science itself, systemizing involves repeating observations in order to establish that the law that has been identified holds true across time and place (excessive “repetitive behaviour”). A “masculinization” of scores on empathy and systemizing tests (i.e., where scores are pushed towards or even beyond the “male range”) is correlated with fetal testosterone levels *in utero* [45–47]. Corresponding with these findings, one of the earliest biological correlates of autism are elevated fetal steroid hormones (not just fetal testosterone but also the precursors to this in the Δ4 sex-steroid pathway, including progesterone, 17α-hydroxyprogesterone, androstenedione, and cortisol) [48].

Although performance on the Eyes test has been shown to be a hallmark of social difficulties in autism, the under-representation of females with autism in previous research makes it difficult to examine whether an individual’s sex further modulates how autism manifests when it comes to social cognitive performance. A large sample-size and a sex-balanced design is needed to examine any hypothesis related to performance comparing males and females with and without autism (e.g., when testing predictions from the EMB theory or other hypotheses about mechanisms and causality, such as sex/gender-differential mechanisms, female protective effect, or better compensation in females, etc. [49]); see [17] for a detailed review. Here we recruited a large, sex-balanced sample to test (1) if there are sex differences and diagnostic differences in performance on the Eyes test, and if there are diagnosis-by-sex interactions, using a two-factorial design [20, 37, 50–52]; and (2) if performance differences between groups conform to predictions from the EMB theory. We were also interested to test if typical sex differences on the Eyes test are simply attenuated, as has been found recently on questionnaire measures [20], or are completely absent. If an absence of sex difference is observed, this might be because on questionnaire measures, females with autism score their empathy traits higher than do males, perhaps associated with unconscious gender stereotypes [53] or a desire to be seen as less impaired. In contrast, performance measures might be less susceptible to such gender stereotypes or social desirability effects, and might reveal cognitive capabilities that are closer to the innate difficulties experienced by individuals with autism.

## Materials and Methods

### Participants and ethics information

Adult participants over 18 years old were recruited from the following websites: www.autismresearchcentre.com (mainly for individuals with autism) or www.cambridgepsychology.com (mainly for individuals without autism), both hosted by the University of Cambridge, during the period of 2007–2014. Once they had logged onto either site, they consented for their data to be held in the Cambridge Autism Research Database (CARD) for research use, with ethical approval (reference No. Pre.2013.06) from the University of Cambridge Psychology Research Ethics Committee. This ethics approval allows for retrospective data analyses, including the present study, in which all participant information were anonymized and de-identified prior to analysis.

Participants were selected from all available participants on the CARD (i.e., everyone who had logged onto the website and completed the Eyes test, AQ and EQ). Participants who self-reported a clinical autism diagnosis were asked specific information about the date of their diagnosis, where they were diagnosed, and the profession of the person who diagnosed them. The inclusion criterion for participants in the autism group was a clinical diagnosis of an autism spectrum condition (ASC) according to DSM-IV (any pervasive developmental disorder), DSM-5 (autism spectrum disorder), or ICD-10 (any pervasive developmental disorder) from a recognized specialist clinic by a psychiatrist or clinical psychologist. Such online self/parent-reported diagnoses agree well with clinical diagnoses in medical records [54]. Control group participants were included if they had no diagnoses of ASC, and no first-degree relatives with ASC. For both groups, participants were excluded if they reported a diagnosis of bipolar disorder, schizophrenia, eating disorder, obsessive-compulsive disorder, personality disorder, epilepsy, or an intersex/transsexual condition. Participants with a diagnosis of depression or anxiety were not excluded as these conditions are common in the general population and occur at high rates in adults with autism [1, 55]. In all four groups (males and females, with or without autism), any participants scoring zero on the Eyes test were removed from analyses, as this could reflect an overall difficulty in understanding test instructions. To minimize the control group including individuals with a broader autism phenotype (BAP) [56, 57], only individuals who had registered via the www.cambridgepsychology.com website, which was designed to recruit participants from the general population, were included in the control group. In addition, because individuals with BAP tend to score above the cut-off of 26 on the Autism Spectrum Quotient (AQ) [56], only a random sample of 6% of males and 2% of females who scored above this cut-off were included in the control group, in line with a non-biased sampling from the general population [56]. In addition, the four groups were selected to match on chronological age. The final sample included 395 adults with autism (178 males, 217 females) and 320 control adults (152 males, 168 females). See Table 1 for demographic and clinical information.

**Table 1:** Demographic and clinical information, AQ, EQ, and Eyes test performance by group.

Group	N	Age	Ethnicity	Rates of depression ^a^	AQ	EQ	Eyes test score	Eyes test-EQ correlation	Eyes test-AQ correlation
		Mean (SD)	% self-described as “White European”	% reporting diagnoses of depression	Mean (SD)	Mean (SD)	Mean (SD)	Pearson’s *r* / Spearman’s *ρ* (2-tailed *p* values)	Pearson’s *r* / Spearman’s *ρ* (2-tailed *p* values)
**Males with autism**	178	39.88 (11.56)	89.3%	33.1%	36.93 (8.64)	18.56 (11.24)	23.53 (6.64)	0.11 / 0.11 (0.162 / 0.132)	-0.13 / -0.14 (0.095 / 0.062)
**Females with autism**	217	39.88 (11.80)	85.3%	40.1%	34.71 (10.97)	24.94 (16.94)	23.45 (7.06)	0.35 / 0.40 (<0.001 / <0.001)	-0.32 / -0.33 (<0.001 / <0.001)
**Control males**	152	37.70 (12.62)	75.7%	9.2%	17.53 (6.03)	41.80 (11.53)	25.54 (4.57)	0.12 / 0.10 (0.132 / 0.201)	-0.13 / -0.16 (0.114 / 0.044)
**Control females**	168	39.15 (10.82)	84.5%	24.4%	15.25 (6.28)	49.88 (12.00)	27.42 (3.43)	0.11 / 0.14 (0.173 / 0.078)	-0.10 / -0.11 (0.190 / 0.157)

N, number; SD, standard deviation

^a^ Participants were inquired whether they have ever been diagnosed with depression; information about anxiety disorders was not specifically collected in the present dataset.

### Measures

The Eyes test (revised and online version) consists of 36 grey-scale photos of people taken from magazines [14, 31]. These photos are cropped and rescaled so that only the area around the eyes can be seen. Each photo is surrounded by four mental state terms and the participant is instructed to choose the word best describes what the person in the photo is thinking or feeling. Only one of the four items is deemed correct (as judged by consensus from an independent panel of judges in the initial psychometric study). Participants were instructed to select the most appropriate item within 20 seconds for each stimulus (presented in random order). The instructions read, “*You are going to see a series of 36 photographs of eyes*. *Your task is to choose the word*, *from a choice of 4*, *that best describes what the person in the picture is thinking or feeling*. *When you think you have found the answer press ‘1’ if it is the top left word*, *‘9’ if it is the top right word*, *‘Q’ if it is the bottom left word and ‘I’ if it is the bottom right word*. *Before making your choice*, *please make sure that you have read all 4 words*. *You should try to answer as quickly as possible*, *but without making mistakes*. *Once you have made a decision the next photograph will appear*. *If you have not made a decision in 20 seconds*, *it will automatically carry on to the next photograph*.” Responses were coded as correct or incorrect (wrong items selected, or no response after 20 seconds), giving a maximum total correct score of 36. All participants also completed the AQ [58] and the EQ [19] on the same online platform. For these self-report questionnaires the instructions read, “*Below are a list of statements*. *Please read each statement very carefully and rate how strongly you agree or disagree with it*.” All participants took the AQ and EQ before taking the Eyes test. The participants could log into and out of their account at any time so did not necessarily take the tests during the same session. However, the order of the tests was fixed for all participants.

### Statistical analysis

To test for differences in total correct score, we used a two-way analysis-of-variance (ANOVA) with diagnosis (autism vs. control) and sex (male vs. female) as the fixed factors. Main and interaction effects were tested at a critical level of α = 0.05. We then compared the performance differences to see if they conformed to the patterns predicted by the EMB theory [41], separately for males and females [50]. Specifically, we tested (1) if there is a typical sex difference between control females and control males (control females > control males); and if yes, (2) whether males with autism show significantly poorer performance than control males; and/or (3) whether females with autism show significantly poorer performance than control females; and lastly, (4) whether any typical sex difference is attenuated in the autism groups.

We also used support vector machine (SVM) classification [59, 60] to test if autism diagnostic status could be accurately classified based on the pattern of responses across the 36 items on the Eyes test (1 = correct, 0 = incorrect). This is to test if patterns of response across items reveal unique information that are not evident in scores that sum correct responses across all 36 items, which would be the case if males and females with autism found different items more difficult. SVM classification was applied to the following pairs of groups: (1) control males and control females, (2) males with and without autism, and (3) females with and without autism. SVM is a supervised multivariate classification method where input data are classified into two classes by identifying a separating hyperplane/decision boundary, which maximizes the margin (i.e., distance from the hyperplane to the nearest data points). The algorithm is initially trained on a subset of the data to find a hyperplane that best separates the input space according to the class labels. Once the decision function is learned from the training set it can be used to predict the class of a new set of test examples. We implemented LIBSVM v3.18 (http://www.csie.ntu.edu.tw/~cjlin/libsvm/) under Matlab R2013b. For the above three within-sample classification tasks, we trained and tested the classifier using a 10-fold cross-validation scheme. To find the classification model we used a radial basis function kernel and a regularization parameter (C) set to the default of 1. During training, class-size imbalance was handled by assigning different weights according to group size. To evaluate performance of the classifier we used the measures of accuracy (percentage of test examples correctly classified) and area under curve (AUC, particularly useful for comparing performance across models derived from imbalanced class-sizes). The performance metrics were tested with 10,000 permutations where the class labels were completely randomized, to evaluate the probability of getting a performance metric higher than the ones obtained during the cross-validation procedure by chance.

Finally, we examined whether the SVM model trained within one sex worked equally well when applied to the other sex, to test if case-control effects are similar or different between sexes. We applied the model classifying males with vs. without autism to the female groups (i.e., out-of-sample data), and vice versa. If case-control effects are different in each sex, a model obtained in one sex should not work well for the other sex; if the case-control effects are similar in both sexes, performance metrics using models obtained from the other sex should be close to the one obtained from within-sample cross-validation.

## Results

### Overall performance on the Eyes test

Performance scores on the Eyes test, EQ and AQ, as well as participant characteristics, are shown in Table 1. A two-way ANOVA showed a significant main effect of diagnosis (F(1,711) = 47.29, *p* < 0.001, η_p_^2^ = 0.062) reflecting better performance in controls than those with autism; and a significant main effect of sex (F(1,711) = 4.284, *p* = 0.039, η_p_^2^ = 0.006), reflecting better performance in females than males. However there was a significant sex-by-diagnosis interaction (F(1,711) = 5.090, *p* = 0.024, η_p_^2^ = 0.007), indicating that the main effects should not be interpreted directly. This interaction was the result from a significant sex difference between control males and control females (*p* < 0.001, Cohen’s *d* = 0.47), but an absence of a sex difference in males and females with autism (*p* = 0.907, *d* = 0.01); meanwhile there were significant case-control differences in both males and females, with a smaller effect size in males (*p* = 0.001, *d* = 0.35) but a larger effect size in females (*p* < 0.001, *d* = 0.69).

**Fig 1:**
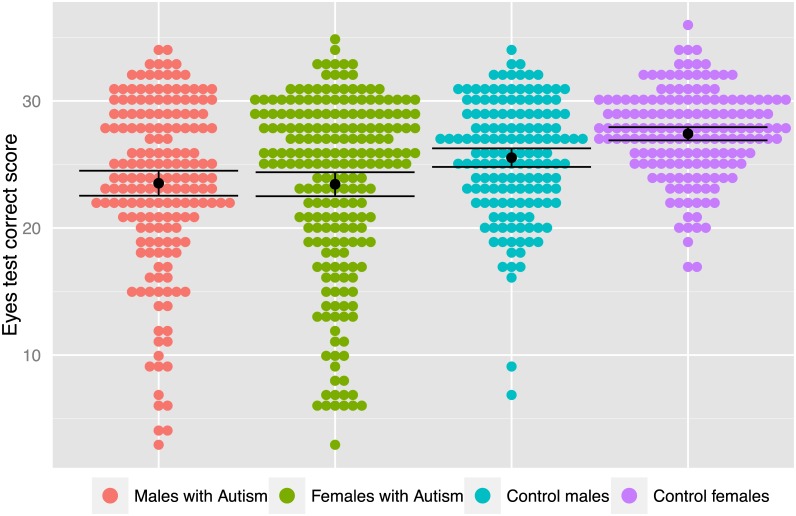
Performance accuracy on the Eyes test by group. Total correct score on the Eyes test for each individual is plotted as a dot, coloured according to group, to illustrate the distribution of performance by group. Mean score for each group is shown by a black dot, and the error bars indicate the 95% confidence interval of the mean score.

Building on the presence of a typical sex difference (control females > control males), diagnostic group differences confirmed predictions from the EMB theory in both males ([control females > control males] AND [control males > males with autism]) and females ([control females > control males] AND [control females > females with autism]) [50]. See Fig 1.

Pearson’s correlation analyses (used here as sample size was large per group and data distribution approximated normality) showed that Eyes test scores were significantly correlated with EQ and AQ scores only in females with autism (Table 1). Fisher’s test [61] showed that the substantial Eyes-EQ correlation in females with autism (*r* = 0.35) was significantly different from that in males with autism (*p* = 0.011), control males (*p* = 0.024), and control females (*p* = 0.013), all *r* ≈ 0.1. The substantial Eyes-AQ correlation in females with autism (*r* = -0.32) was also significantly different from that in males with autism (*p* = 0.042), control males (*p* = 0.057), and control females (*p* = 0.026), all *r* ≈ -0.1. As data distributions were slightly skewed in the autism groups, non-parametric Spearman’s correlations were also performed, and the same group-differential correlation patterns were confirmed (Table 1).

### Group classification based on response patterns on the Eyes test

SVM classification with 10-fold cross-validation showed that the model classified control males vs. control females with modest performance, and significantly better than chance using permutation testing (accuracy 54.7%, *p* = 9.99 × 10^−5^; AUC 0.568, *p* = 9.99 × 10^−5^). The classification between control males vs. males with autism was better and significantly better than chance (accuracy 65.8%, *p* = 9.99 × 10^−5^; AUC 0.678, *p* = 9.99 × 10^−5^), and the classification between control females vs. females with autism was even better (accuracy 72.2%, *p* = 9.99 × 10^−5^; AUC 0.729, *p* = 9.99 × 10^−5^). Using the model obtained from classifying diagnostic status in males to predict diagnostic status in females showed accuracy (70.9%) close to the within-female classification using cross-validation (72.2%). Similarly, using the model from females to predict diagnostic status in males showed accuracy (65.2%) close to that obtained by the within-male classification using cross-validation (65.8%). These together suggest that in terms of response pattern, case-control effects are similar across both sexes.

## Discussion

In an age-matched, non-male-biased large sample of adults with and without autism, we tested Eyes test performance to establish if there were (1) typical sex differences, (2) differences between diagnostic groups, (3) an interaction of these, and (4) if the pattern of group differences fitted predictions from the EMB theory. We confirmed previous results showing a typical sex difference [14], with control females scoring higher than control males. We also confirmed previous results showing that, in both males and females, individuals with autism score significantly lower than typically developing controls [16, 34–38]. Furthermore, and importantly, we found that sex and diagnosis significantly modulate each other: the diagnostic effect was much larger in females than in males, and the typical sex difference was completely absent in the autism groups. The patterns of group differences fit predictions from the EMB theory in both males (i.e., [control females > control males] AND [control males > males with autism]) and females (i.e., [control females > control males] AND [control females > females with autism]) [50]. Finally, when examining response patterns, effects of diagnostic group were similar across males and females.

### Difficulty in mental state attribution and complex facial emotion recognition from the eye region is found in both males and females with autism

Performance on the Eyes test is a reliable [32, 33, 62] phenotypic [16, 34–38] and endophenotypic [35, 63, 64] measure for the cognitive bases of autism. How such phenotypic and endophenotypic characteristics are further modulated by sex has not previously been examined in detail. The present findings of similar performance between adult males and females with autism, and the larger shift in females than in males with autism away from same-sex controls, replicates our earlier findings from a smaller adult sample [37]. In addition, the classification model based on item response patterns obtained within one sex appears to work equally well when applied to the other sex. Together, these findings confirm the validity of the Eyes test as a *sex-independent* phenotypic measure of autism, indicating mental state attribution and complex facial emotion recognition are aspects of the core social difficulties in both males and females with autism.

Typically developing adults perform just as well on complex emotion/mental state recognition when the eyes alone are the only cue, or when the whole face is available [65]. This suggests that the eyes alone contain sufficient information as the whole face does, during complex emotion recognition. In contrast, individuals with autism show poorer performance on complex emotion/mental state recognition by the eyes alone than by the whole face, and perform significantly worse than age- and IQ-matched controls at identifying complex emotion/mental states from both the eyes alone and the whole face conditions [65]. This suggests that there may be a “language of the eyes” [65–68] that we use ToM to identify other people’s mental state from their eyes, and that individuals with autism are weaker on this.

The difficulties that adults and adolescents with autism have on the Eyes test [14, 36] mirror more basic difficulties earlier in development, using eye direction as a cue that someone is thinking [69], to infer a person’s desires and goals [70], or to infer which object is the intended reference to decode speech during language acquisition [71]. Reduced attention to the eye region of the face is one of the earliest abnormalities in autism developmentally [72–74]. It may not only be an early infancy marker of the later difficulties in ToM/cognitive empathy, but also a contributor to it [75], as it may result in reduced social orientation, social reward and social learning, and increased risk for a later autism diagnosis and social disability [76, 77].

### Group-specific correlation between Eyes test performance and self-report dispositional traits

In previous studies conducted in the general population and not sex-stratified, Eyes test scores show an inverse correlation with AQ scores [58, 78], and a positive correlation with EQ scores [78]. When sex-stratified, a university student-based mid-sized study (65 men and 79 women) showed a significant negative AQ-Eyes test correlation only in men but not in women [79]. Interestingly, in the present large-scale study, when analyses were stratified by both sex and diagnosis, the correlation patterns between Eyes test performance and self-reported empathy/autistic traits were found to be both sex- and diagnosis-dependent. Eyes-EQ and Eyes-AQ correlations were significant *only* in females with autism, but not in males with autism, control males, or control females. The correlations in females with autism were statistically significantly different from those in the other three groups, suggesting stronger coherence between self-reflected/evaluated traits and neuropsychological performance in females with autism. In the general population, self-reported autistic traits are often associated with social cognitive performance in males but not in females, suggesting a more fractionable neurocognitive structure in females, potentially indicative of a “female protective effect” [17]. The reversal was found here for adults with autism, perhaps reflecting a loss of such fractionation in females with autism. Another possibility may be that adult females with autism have heightened self-awareness (possibly affected by socio-cultural contexts [80]), reflected in the significant association between their objective mentalizing performance and their subjective reflection of their personal empathy-related traits. These hypotheses are speculative and await further investigation. Whether this female-specific pattern of heightened association between self-reflected/evaluated traits and social cognitive performance are associated with a plausible “female-phenotype” of autism that has been anecdotally reported (e.g., heightened social awareness and social motivation, better imitation, more camouflaging) [17] is an important focus for further investigation.

### Confirmation of EMB theory predictions in both males and females

Predictions from the EMB theory on Eyes test performance were confirmed in both males (i.e., [control females > control males] AND [control males > males with autism]) and females (i.e., [control females > control males] AND [control females > females with autism]). In addition, unlike attenuation of typical sex difference in autism found on self-report traits [20], here we found a complete absence of typical sex difference on the Eyes test. This may partly be because performance measures are less susceptible than self-report questionnaires to implicit gender stereotypes or social desirability effects, and reflect ability-based characteristics. It is also interesting to note that the EMB theory predictions were clearer in females than in males with autism, as shown by the larger case-control differences in the female than the male groups. These might be because the “masculinization” effects/characteristics of autism are more readily observable in females than males with autism, as previously noted in other domains including childhood play [81], brain structural characteristics [50], serum hormonal level [82–84], and anthropometry [84].

### Potential uses of the Eyes test

The Eyes test has been used in both neuroimaging and lesion studies, revealing the involvement of the inferior frontal and temporal gyri, and amygdala [85–87]. During repeat fMRI between ages 12–19 years old, activation of the right superior temporal sulcus and right inferior frontal gyrus for the contrast “mental state > control” is a stable pattern of activity in performing the Eyes test [88]. However, partially sex-dependent functional brain correlates during performance on the Eyes test have been found in adolescents with and without autism [36]. Interestingly, neuro-endophenotypic effects are also present, and are again stronger in females [36]. Performance on the Eyes test among typically developing boys and girls shows an inverse association with prenatal testosterone levels [45], and in adulthood shows association with single nucleotide polymorphisms in *OXTR* [89], *NTRK2*, *NTRK3*, *HSD17B2*, *HSD17B4*, *CYP1B1*, *CYP7A1*, *EN2*, and *GABRA6* [90]. Argenine-vasopressin administration in typically developing males reduces performance on the Eyes test, compared to placebo [91]. Similarly, testosterone administration in typically developing females reduces performance on the Eyes test, compared to placebo [92]. Administration of 3,4-methylenedioxymethamphetamine (MDMA, or ecstasy) enhances recognition of positive emotions but impairs recognition of negative emotions on the Eyes test across both general population males and females [93]. On the other hand, oxytocin administration in males with autism improves performance on the Eyes test, compared to placebo [94]. Future studies need to identify sex-common and sex-specific cognitive, neurobiological and psychopharmacological correlates of the Eyes test, and related tasks measuring cognitive vs. affective empathy, in individuals with autism or other atypical social-affective developmental conditions across the lifespan.

Performance on the Eyes test also reveals individual differences within the general population. It has been found to differentiate “high tech” (e.g., surgeons) vs. “high touch” (e.g., psychiatrists) doctors [95]. As mentioned above, it has also been used as a sensitive outcome measure for oxytocin administration studies [96, 97]. Finally, in terms of clinical groups, individuals with psychopathy [98], social anxiety disorder [99], schizophrenia [100], borderline personality disorder [101, 102], or victims of child abuse and neglect [103] all show different patterns of atypical performance on the Eyes test. Women with anorexia have been postulated to include individuals with undiagnosed autism, and show similar impairment on the Eyes test to people with a formal diagnosis of autism [104–106]. How such performance is modulated by sex requires further investigation.

The SVM classification between control males vs. control females was only modest (accuracy 54.7%, AUC 0.568). This shows that performance on the Eyes test, whilst sex-linked, is not wholly determined by one’s sex. One might need a battery of tests to better classify typically developing males vs. females. Even then, we suspect that measures such as the Eyes test would better classify cognitive styles [107] than sex. In addition, both classification performance between control males vs. males with autism (accuracy 65.8%, AUC 0.678) and between control females vs. females with autism (accuracy 72.2%, AUC 0.729), though significant, were not accurate enough to be diagnostic when used in isolation. This is not surprising, as the Eyes test is only related to part of the autism phenotype, and there are other aspects of social cognition, as well as executive, visuo-spatial, and sensori-perceptual features that are linked to the autism phenotype. Thus, we would need a combination of cognitive tasks to improve the accuracy of classifying autism vs. controls, and to potentially help clinical diagnostic procedures [38]. Nevertheless, cognitive performance still reveals individual differences and the heterogeneity of the autism spectrum [108, 109], reflected in the substantial inter-individual variation in the present study (Fig 1). This may be useful in the context of individualized education and medicine.

### Limitations

Some limitations of the present study should be acknowledged. First, Eyes test performance has been associated with verbal IQ [110] or even performance IQ [111]. This may reflect that on this task, mental state recognition requires selecting one out of four mental state words, and a person’s mental state lexicon may itself be related to their verbal or performance IQ. In the present study we have no measure of verbal IQ or language performance, nor performance IQ, so cannot test the extent to which the present findings are influenced by intelligence and linguistic abilities. Future studies taking into account variances explained by measured IQ or linguistic abilities are needed to further clarify group difference patterns seen in the present study.

Second, this study only included (higher-functioning) individuals who could complete online tasks and self-reported their formal clinical diagnoses of autism, and we further excluded those who had significant co-occurring psychiatric conditions. We therefore do not know if the findings would generalize to subgroups with intellectual disabilities, those with significant psychiatric comorbidity, or those without access to the internet or who are unable to volunteer for online research.

Third, it has been shown that Eyes test performance can be influenced by the correspondence between the ethnicity of the participant and that of the stimuli used in the test [112]. Our stimuli are all taken from Caucasian faces and the majority of participants across four groups self-describe as “White European”. However the very small numbers and the wide distribution of participants reporting other ethnicities renders a statistically sound examination of potential moderating effects from ethnicity and culture (and potential effects of familiarity) difficult. The extent to which current findings may be modulated by ethnic, cultural, or familiarity factors remains to be clarified by large-scale cross-ethnic and cross-cultural datasets.

Fourth, we were not able to investigate the complex interactions between affect regulation/emotion disorders (which frequently co-occur with autism) and how sex and autism diagnosis influence Eyes test performance in the present study, because we did not collect data for anxiety disorders (which frequently co-occur with depression), and also because we lack the statistical power to address complex three-way interactions (between emotion disorders, sex, and autism). The extent to which the present findings may be affected by co-occurring affect regulation difficulties is therefore unknown. This is an important topic to investigate in the future, with a better-powered and more comprehensive dataset.

Fifth, there was no independent in-person verification of diagnoses for the majority of the autism groups since participants were recruited online. However, previous studies have shown high levels of agreement between self/parent-reported diagnoses and clinical diagnoses of autism in medical records [54]. In addition, all participants with autism provided the name of the psychiatrist or clinical psychologist who had diagnosed them and the name of the clinic where they were diagnosed, and we have no obvious reason to disbelieve such data.

Finally, the present study is cross-sectional and only about cognitive characteristics. Therefore the study is only at the phenotypic and descriptive level and demonstrates differences and similarities between males and females with ASC. Other hypotheses about mechanisms and causality in relation to sex/gender differences in autism, such as sex/gender-differential mechanisms, female protective effects, or better compensation in females [49] need to be tested with approaches that can reveal developmental mechanisms and etiologies, and that use longitudinal and/or multi-level datasets. In addition, for such hypothesis testing, clinical phenotypes need to be measured comprehensively in order for any cross-sex/gender comparison to be unambiguously interpreted [17]. The present study is not able to test for etiologies or developmental mechanisms, but is a useful foundation for phenotypic characterization of autism, taking sex and gender into account.

## Conclusions

In a large, non-male-biased adult sample, we confirm that performance (in terms of both accuracy and response pattern) on the Eyes test is a sex-independent phenotypic characteristic of individuals with autism, reflecting sex-common social difficulties. The presence of a typical sex difference in performance on the Eyes test confirms previous reports of a female advantage in cognitive empathy. Performance of females with autism deviated away from same-sex controls to a greater extent than that seen in males with autism. Females with autism also showed stronger coherence between self-reported dispositional traits and Eyes test performance than all other groups. Finally, the findings provide support to predictions from the EMB theory, in both sexes, in terms of performance accuracy.

## References

[1] Lai MC, Lombardo MV, Baron-Cohen S. Autism. Lancet. 2014;383(9920):896–910. Epub 2013/10/01. 10.1016/S0140-6736(13)61539-1 . 24074734

[2] American Psychiatric Association. Diagnostic and Statistical Manual of Mental Disorders, 5th edition (DSM-5). Washington, DC: American Psychiatric Publishing, Inc.; 2013.

[3] Baron-Cohen S. Mindblindness: An essay on autism and theory of mind. Boston: MIT Press/Bradford Books; 1995.

[4] Whiten A. Natural theories of mind: Evolution, development and simulation of everyday mindreading: Basil Blackwell Oxford; 1991.

[5] Frith U, Morton J, Leslie AM. The cognitive basis of a biological disorder: autism. Trends Neurosci. 1991;14(10):433–8. . 1722361 10.1016/0166-2236(91)90041-r

[6] Lombardo MV, Baron-Cohen S. The role of the self in mindblindness in autism. Conscious Cogn. 2011;20(1):130–40. Epub 2010/10/12. doi: S1053-8100(10)00172-8 [pii] 10.1016/j.concog.2010.09.006 . 20932779

[7] Baron‐Cohen S. Perceptual role taking and protodeclarative pointing in autism. British Journal of Developmental Psychology. 1989;7(2):113–27.

[8] Mundy P, Sigman M, Ungerer J, Sherman T. Defining the social deficits of autism: the contribution of non-verbal communication measures. J Child Psychol Psychiatry. 1986;27(5):657–69. . 3771682 10.1111/j.1469-7610.1986.tb00190.x

[9] Baron‐Cohen S. Autism and symbolic play. British Journal of Developmental Psychology. 1987;5(2):139–48.

[10] Baron-Cohen S, Leslie AM, Frith U. Does the autistic child have a "theory of mind"? Cognition. 1985;21(1):37–46. . 2934210 10.1016/0010-0277(85)90022-8

[11] Happe FG. The role of age and verbal ability in the theory of mind task performance of subjects with autism. Child Dev. 1995;66(3):843–55. . 7789204

[12] Baron-Cohen S. The autistic child's theory of mind: a case of specific developmental delay. J Child Psychol Psychiatry. 1989;30(2):285–97. . 2523408 10.1111/j.1469-7610.1989.tb00241.x

[13] Baron-Cohen S, O'Riordan M, Stone V, Jones R, Plaisted K. Recognition of faux pas by normally developing children and children with Asperger syndrome or high-functioning autism. J Autism Dev Disord. 1999;29(5):407–18. . 10587887 10.1023/a:1023035012436

[14] Baron-Cohen S, Wheelwright S, Hill J, Raste Y, Plumb I. The "Reading the Mind in the Eyes" Test revised version: a study with normal adults, and adults with Asperger syndrome or high-functioning autism. J Child Psychol Psychiatry. 2001;42(2):241–51. . 11280420

[15] Rutherford MD, Baron-Cohen S, Wheelwright S. Reading the mind in the voice: a study with normal adults and adults with Asperger syndrome and high functioning autism. J Autism Dev Disord. 2002;32(3):189–94. . 12108620 10.1023/a:1015497629971

[16] Golan O, Baron-Cohen S. Systemizing empathy: teaching adults with Asperger syndrome or high-functioning autism to recognize complex emotions using interactive multimedia. Dev Psychopathol. 2006;18(2):591–617. . 16600069 10.1017/S0954579406060305

[17] Lai MC, Lombardo MV, Auyeung B, Chakrabarti B, Baron-Cohen S. Sex/gender differences and autism: setting the scene for future research. J Am Acad Child Adolesc Psychiatry. 2015;54(1):11–24. 10.1016/j.jaac.2014.10.003 25524786 PMC4284309

[18] Senju A. Spontaneous theory of mind and its absence in autism spectrum disorders. Neuroscientist. 2012;18(2):108–13. Epub 2011/05/26. 10.1177/1073858410397208 . 21609942 PMC3796729

[19] Baron-Cohen S, Wheelwright S. The Empathy Quotient: an investigation of adults with Asperger syndrome or high functioning autism, and normal sex differences. J Autism Dev Disord. 2004;34(2):163–75. . 15162935 10.1023/b:jadd.0000022607.19833.00

[20] Baron-Cohen S, Cassidy S, Auyeung B, Allison C, Achoukhi M, Robertson S, et al Attenuation of typical sex differences in 800 adults with autism vs. 3,900 controls. PLoS ONE. 2014;9(7):e102251 10.1371/journal.pone.0102251 25029203 PMC4100876

[21] Auyeung B, Wheelwright S, Allison C, Atkinson M, Samarawickrema N, Baron-Cohen S. The children's Empathy Quotient and Systemizing Quotient: sex differences in typical development and in Autism Spectrum Conditions. J Autism Dev Disord. 2009;39(11):1509–21. Epub 2009/06/18. 10.1007/s10803-009-0772-x . 19533317

[22] Davis MH. Empathy: A social psychological approach: Westview Press; 1994.

[23] Baron-Cohen S, Tager-Flusberg H, Lombardo MV. Understanding other minds: Perspectives from developmental social neuroscience. Oxford, UK: Oxford University Press; 2013.

[24] Rogers K, Dziobek I, Hassenstab J, Wolf OT, Convit A. Who cares? Revisiting empathy in Asperger syndrome. J Autism Dev Disord. 2007;37(4):709–15. 10.1007/s10803-006-0197-8 . 16906462

[25] Bird G, Cook R. Mixed emotions: the contribution of alexithymia to the emotional symptoms of autism. Translational psychiatry. 2013;3:e285 Epub 2013/07/25. 10.1038/tp.2013.61 tp201361 [pii]. . 23880881 PMC3731793

[26] Baron-Cohen S. Zero degrees of empathy: A new theory of human cruelty: Penguin UK; 2011. 10.2989/17280583.2012.735509 25860184

[27] Decety J, Skelly LR, Kiehl KA. Brain response to empathy-eliciting scenarios involving pain in incarcerated individuals with psychopathy. JAMA psychiatry. 2013;70(6):638–45. 10.1001/jamapsychiatry.2013.27 23615636 PMC3914759

[28] Decety J, Chen C, Harenski C, Kiehl KA. An fMRI study of affective perspective taking in individuals with psychopathy: imagining another in pain does not evoke empathy. Frontiers in human neuroscience. 2013;7:489 10.3389/fnhum.2013.00489 24093010 PMC3782696

[29] Blair RJ, Jones L, Clark F, Smith M. The psychopathic individual: a lack of responsiveness to distress cues? Psychophysiology. 1997;34(2):192–8. . 9090269 10.1111/j.1469-8986.1997.tb02131.x

[30] Mazza M, Pino MC, Mariano M, Tempesta D, Ferrara M, De Berardis D, et al Affective and cognitive empathy in adolescents with autism spectrum disorder. Front Hum Neurosci. 2014;8:791 10.3389/fnhum.2014.00791 25339889 PMC4187579

[31] Baron-Cohen S, Jolliffe T, Mortimore C, Robertson M. Another advanced test of theory of mind: evidence from very high functioning adults with autism or asperger syndrome. J Child Psychol Psychiatry. 1997;38(7):813–22. . 9363580 10.1111/j.1469-7610.1997.tb01599.x

[32] Fernandez-Abascal EG, Cabello R, Fernandez-Berrocal P, Baron-Cohen S. Test-retest reliability of the 'Reading the Mind in the Eyes' test: a one-year follow-up study. Mol Autism. 2013;4(1):33 10.1186/2040-2392-4-33 24020728 PMC3848772

[33] Vellante M, Baron-Cohen S, Melis M, Marrone M, Petretto DR, Masala C, et al The "Reading the Mind in the Eyes" test: systematic review of psychometric properties and a validation study in Italy. Cogn Neuropsychiatry. 2013;18(4):326–54. Epub 2012/10/31. 10.1080/13546805.2012.721728 . 23106125 PMC6345369

[34] Lombardo MV, Barnes JL, Wheelwright SJ, Baron-Cohen S. Self-referential cognition and empathy in autism. PLoS ONE. 2007;2(9):e883 Epub 2007/09/13. 10.1371/journal.pone.0000883 . 17849012 PMC1964804

[35] Losh M, Adolphs R, Poe MD, Couture S, Penn D, Baranek GT, et al Neuropsychological profile of autism and the broad autism phenotype. Arch Gen Psychiatry. 2009;66(5):518–26. Epub 2009/05/06. doi: 66/5/518 [pii] 10.1001/archgenpsychiatry.2009.34 19414711 PMC2699548

[36] Holt RJ, Chura LR, Lai MC, Suckling J, von dem Hagen EA, Calder AJ, et al ‘Reading the Mind in the Eyes’: an fMRI study of adolescents with autism and their siblings. Psychol Med. 2014 10.1017/S0033291714000233 PMC6345365 25065819

[37] Lai MC, Lombardo MV, Ruigrok AN, Chakrabarti B, Wheelwright SJ, Auyeung B, et al Cognition in males and females with autism: similarities and differences. PLoS ONE. 2012;7(10):e47198 Epub 2012/10/25. 10.1371/journal.pone.0047198 23094036 PMC3474800

[38] Wilson CE, Happe F, Wheelwright SJ, Ecker C, Lombardo MV, Johnston P, et al The neuropsychology of male adults with high-functioning autism or asperger syndrome. Autism Res. 2014;7(5):568–81. 10.1002/aur.1394 . 24903974 PMC4489335

[39] Schiffer B, Pawliczek C, Muller BW, Gizewski ER, Walter H. Why don't men understand women? Altered neural networks for reading the language of male and female eyes. PLoS One. 2013;8(4):e60278 10.1371/journal.pone.0060278 23593185 PMC3622659

[40] Rutherford HJ, Wareham JD, Vrouva I, Mayes LC, Fonagy P, Potenza MN. Sex differences moderate the relationship between adolescent language and mentalization. Personality disorders. 2012;3(4):393–405. 10.1037/a0028938 22800178 PMC3691855

[41] Baron-Cohen S. The extreme male brain theory of autism. Trends Cogn Sci. 2002;6(6):248–54. . 12039606 10.1016/s1364-6613(02)01904-6

[42] Baron-Cohen S. The essential difference. New York, NY: Basic Books; 2003.

[43] Baron-Cohen S. Two new theories of autism: hyper-systemising and assortative mating. Arch Dis Child. 2006;91(1):2–5. . 16371371 10.1136/adc.2005.075846 PMC2083102

[44] Eisenberg L, Kanner L. Childhood schizophrenia; symposium, 1955. VI. Early infantile autism, 1943–55. Am J Orthopsychiatry. 1956;26(3):556–66. . 13339939 10.1111/j.1939-0025.1956.tb06202.x

[45] Chapman E, Baron-Cohen S, Auyeung B, Knickmeyer R, Taylor K, Hackett G. Fetal testosterone and empathy: evidence from the Empathy Quotient (EQ) and the "Reading the Mind in the Eyes" test. Soc Neurosci. 2006;1(2):135–48. Epub 2008/07/18. doi: 759346795 [pii] 10.1080/17470910600992239 . 18633782

[46] Auyeung B, Baron-Cohen S, Ashwin E, Knickmeyer R, Taylor K, Hackett G, et al Fetal testosterone predicts sexually differentiated childhood behavior in girls and in boys. Psychol Sci. 2009;20(2):144–8. Epub 2009/01/30. doi: PSCI2279 [pii] 10.1111/j.1467-9280.2009.02279.x 19175758 PMC2778233

[47] Auyeung B, Baron-Cohen S, Ashwin E, Knickmeyer R, Taylor K, Hackett G. Fetal testosterone and autistic traits. Br J Psychol. 2009;100(Pt 1):1–22. Epub 2008/06/13. doi: 298715 [pii] 10.1348/000712608X311731 . 18547459

[48] Baron-Cohen S, Auyeung B, Norgaard-Pedersen B, Hougaard DM, Abdallah MW, Melgaard L, et al Elevated fetal steroidogenic activity in autism. Mol Psychiatry. 2015;20(3):369–76. 10.1038/mp.2014.48 24888361 PMC4184868

[49] Lai MC, Lombardo MV, Pasco G, Ruigrok AN, Wheelwright SJ, Sadek SA, et al A behavioral comparison of male and female adults with high functioning autism spectrum conditions. PLoS ONE. 2011;6(6):e20835 10.1371/journal.pone.0020835 PONE-D-11-03038 [pii]. . 21695147 PMC3113855

[50] Lai MC, Lombardo MV, Suckling J, Ruigrok AN, Chakrabarti B, Ecker C, et al Biological sex affects the neurobiology of autism. Brain. 2013;136(Pt 9):2799–815. Epub 2013/08/13. 10.1093/brain/awt216 23935125 PMC3754459

[51] Beacher FD, Minati L, Baron-Cohen S, Lombardo MV, Lai MC, Gray MA, et al Autism attenuates sex differences in brain structure: a combined voxel-based morphometry and diffusion tensor imaging study. AJNR Am J Neuroradiol. 2012;33(1):83–9. Epub 2011/12/17. doi: ajnr.A2880 [pii] 10.3174/ajnr.A2880 . 22173769 PMC6345364

[52] Beacher FD, Radulescu E, Minati L, Baron-Cohen S, Lombardo MV, Lai MC, et al Sex differences and autism: Brain function during verbal fluency and mental rotation. PLoS ONE. 2012;7(6):e38355 Epub 2012/06/16. 10.1371/journal.pone.0038355 22701630 PMC3373504

[53] Sinclair S, Hardin CD, Lowery BS. Self-stereotyping in the context of multiple social identities. J Pers Soc Psychol. 2006;90(4):529–42. 10.1037/0022-3514.90.4.529 . 16649853

[54] Daniels AM, Rosenberg RE, Anderson C, Law JK, Marvin AR, Law PA. Verification of parent-report of child autism spectrum disorder diagnosis to a web-based autism registry. J Autism Dev Disord. 2012;42(2):257–65. 10.1007/s10803-011-1236-7 . 21468770

[55] Cassidy S, Bradley P, Robinson J, Allison C, McHugh M, Baron-Cohen S. Suicidal ideation and suicide plans or attempts in adults with Asperger’s syndrome attending a specialist diagnostic clinic: a clinical cohort study. Lancet Psychiatry. 2014;1(2):142–7. 10.1016/S2215-0366(14)70248-2 26360578

[56] Wheelwright S, Auyeung B, Allison C, Baron-Cohen S. Defining the broader, medium and narrow autism phenotype among parents using the Autism Spectrum Quotient (AQ). Mol Autism. 2010;1(1):10. Epub 2010/08/04. doi: 2040-2392-1-10 [pii] 10.1186/2040-2392-1-10 20678260 PMC2913943

[57] Piven J, Palmer P, Jacobi D, Childress D, Arndt S. Broader autism phenotype: evidence from a family history study of multiple-incidence autism families. Am J Psychiatry. 1997;154(2):185–90. . 9016266 10.1176/ajp.154.2.185

[58] Baron-Cohen S, Wheelwright S, Skinner R, Martin J, Clubley E. The Autism Spectrum Quotient (AQ): evidence from Asperger syndrome/high-functioning autism, males and females, scientists and mathematicians. J Autism Dev Disord. 2001;31(1):5–17. . 11439754 10.1023/a:1005653411471

[59] Burges CJ. A tutorial on support vector machines for pattern recognition. Data mining and knowledge discovery. 1998;2(2):121–67.

[60] Schoelkopf B, Smola A. Learning with Kernels. MIT Press, Cambridge, MA; 2002.

[61] Fisher RA. On the probable error of a coefficient of correlation deduced from a small sample. Metron. 1921;1:3–32.

[62] Prevost M, Carrier ME, Chowne G, Zelkowitz P, Joseph L, Gold I. The Reading the Mind in the Eyes test: validation of a French version and exploration of cultural variations in a multi-ethnic city. Cognitive neuropsychiatry. 2014;19(3):189–204. 10.1080/13546805.2013.823859 . 23937473

[63] Dorris L, Espie CA, Knott F, Salt J. Mind-reading difficulties in the siblings of people with Asperger's syndrome: evidence for a genetic influence in the abnormal development of a specific cognitive domain. J Child Psychol Psychiatry. 2004;45(2):412–8. . 14982254 10.1111/j.1469-7610.2004.00232.x

[64] Baron-Cohen S, Hammer J. Parents of children with Asperger Syndrome: What is the cognitive phenotype? J Cogn Neurosci. 1997;9(4):548–54. 10.1162/jocn.1997.9.4.548 . 23968217

[65] Baron-Cohen S, Wheelwright S, Jolliffe T. Is there a" language of the eyes"? Evidence from normal adults, and adults with autism or Asperger syndrome. Visual Cognition. 1997;4(3):311–31.

[66] Wyk BC, Hudac CM, Carter EJ, Sobel DM, Pelphrey KA. Action understanding in the superior temporal sulcus region. Psychol Sci. 2009;20(6):771–7. 10.1111/j.1467-9280.2009.02359.x 19422619 PMC2849148

[67] Senju A, Johnson MH. The eye contact effect: mechanisms and development. Trends Cogn Sci. 2009;13(3):127–34. 10.1016/j.tics.2008.11.009 . 19217822

[68] Carlin JD, Calder AJ. The neural basis of eye gaze processing. Curr Opin Neurobiol. 2013;23(3):450–5. 10.1016/j.conb.2012.11.014 . 23266245

[69] Baron-Cohen S, Cross P. Reading the eyes: Evidence for the role of perception in the development of a theory of mind. Mind & Language. 1992;7(1‐2):172–86.

[70] Baron‐Cohen S, Campbell R, Karmiloff‐Smith A, Grant J, Walker J. Are children with autism blind to the mentalistic significance of the eyes? British Journal of Developmental Psychology. 1995;13(4):379–98.

[71] Baron-Cohen S, Baldwin DA, Crowson M. Do children with autism use the speaker's direction of gaze strategy to crack the code of language? Child Dev. 1997;68(1):48–57. . 9084124

[72] Jones W, Klin A. Attention to eyes is present but in decline in 2-6-month-old infants later diagnosed with autism. Nature. 2013;504(7480):427–31. 10.1038/nature12715 24196715 PMC4035120

[73] Elsabbagh M, Gliga T, Pickles A, Hudry K, Charman T, Johnson MH, et al The development of face orienting mechanisms in infants at-risk for autism. Behav Brain Res. 2013;251:147–54. 10.1016/j.bbr.2012.07.030 22846849 PMC3730054

[74] Baron-Cohen S, Cox A, Baird G, Swettenham J, Nightingale N, Morgan K, et al Psychological markers in the detection of autism in infancy in a large population. Br J Psychiatry. 1996;168(2):158–63. . 8837904 10.1192/bjp.168.2.158

[75] Boucher J. Putting theory of mind in its place: psychological explanations of the socio-emotional-communicative impairments in autistic spectrum disorder. Autism. 2012;16(3):226–46. Epub 2012/02/03. doi: 1362361311430403 [pii] 10.1177/1362361311430403 . 22297199

[76] Jones W, Carr K, Klin A. Absence of preferential looking to the eyes of approaching adults predicts level of social disability in 2-year-old toddlers with autism spectrum disorder. Arch Gen Psychiatry. 2008;65(8):946–54. Epub 2008/08/06. doi: 65/8/946 [pii] 10.1001/archpsyc.65.8.946 . 18678799

[77] Chawarska K, Shic F, Macari S, Campbell DJ, Brian J, Landa R, et al 18-month predictors of later outcomes in younger siblings of children with autism spectrum disorder: a baby siblings research consortium study. J Am Acad Child Adolesc Psychiatry. 2014;53(12):1317–27 e1 10.1016/j.jaac.2014.09.015 25457930 PMC4254798

[78] Voracek M, Dressler SG. Lack of correlation between digit ratio (2D: 4D) and Baron-Cohen’s “Reading the Mind in the Eyes” test, empathy, systemising, and autism-spectrum quotients in a general population sample. Personality and Individual Differences. 2006;41(8):1481–91.

[79] Valla JM, Ganzel BL, Yoder KJ, Chen GM, Lyman LT, Sidari AP, et al More than maths and mindreading: sex differences in empathizing/systemizing covariance. Autism Res. 2010;3(4):174–84. Epub 2010/07/01. 10.1002/aur.143 . 20589713

[80] Kreiser NL, White SW. ASD in females: Are we overstating the gender difference in diagnosis? Clinical child and family psychology review. 2014;17(1):67–84. Epub 2013/07/10. 10.1007/s10567-013-0148-9 . 23836119

[81] Knickmeyer RC, Wheelwright S, Baron-Cohen S. Sex-typical play: masculinization/defeminization in girls with an autism spectrum condition. J Autism Dev Disord. 2008;38(6):1028–35. Epub 2007/11/07. 10.1007/s10803-007-0475-0 . 17985222

[82] Ruta L, Ingudomnukul E, Taylor K, Chakrabarti B, Baron-Cohen S. Increased serum androstenedione in adults with autism spectrum conditions. Psychoneuroendocrinology. 2011;36(8):1154–63. Epub 2011/03/15. doi: S0306-4530(11)00059-X [pii] 10.1016/j.psyneuen.2011.02.007 . 21398041

[83] Schwarz E, Guest PC, Rahmoune H, Wang L, Levin Y, Ingudomnukul E, et al Sex-specific serum biomarker patterns in adults with Asperger's syndrome. Mol Psychiatry. 2011;16(12):1213–20. Epub 2010/09/30. 10.1038/mp.2010.102 mp2010102 [pii]. . 20877284

[84] Bejerot S, Eriksson JM, Bonde S, Carlstrom K, Humble MB, Eriksson E. The extreme male brain revisited: gender coherence in adults with autism spectrum disorder. Br J Psychiatry. 2012;201:116–23. Epub 2012/04/14. 10.1192/bjp.bp.111.097899 . 22500012

[85] Dal Monte O, Schintu S, Pardini M, Berti A, Wassermann EM, Grafman J, et al The left inferior frontal gyrus is crucial for reading the mind in the eyes: Brain lesion evidence. Cortex. 2014;58:9–17. 10.1016/j.cortex.2014.05.002 . 24946302

[86] Baron-Cohen S, Ring HA, Wheelwright S, Bullmore ET, Brammer MJ, Simmons A, et al Social intelligence in the normal and autistic brain: an fMRI study. Eur J Neurosci. 1999;11(6):1891–8. . 10336657 10.1046/j.1460-9568.1999.00621.x

[87] Stone VE, Baron-Cohen S, Calder A, Keane J, Young A. Acquired theory of mind impairments in individuals with bilateral amygdala lesions. Neuropsychologia. 2003;41(2):209–20. . 12459219 10.1016/s0028-3932(02)00151-3

[88] Overgaauw S, van Duijvenvoorde AC, Gunther Moor B, Crone EA. A longitudinal analysis of neural regions involved in reading the mind in the eyes. Soc Cogn Affect Neurosci. 2014 10.1093/scan/nsu095 . 25062837 PMC4420738

[89] Rodrigues SM, Saslow LR, Garcia N, John OP, Keltner D. Oxytocin receptor genetic variation relates to empathy and stress reactivity in humans. Proceedings of the National Academy of Sciences of the United States of America. 2009;106(50):21437–41. 10.1073/pnas.0909579106 19934046 PMC2795557

[90] Chakrabarti B, Dudbridge F, Kent L, Wheelwright S, Hill-Cawthorne G, Allison C, et al Genes related to sex steroids, neural growth, and social-emotional behavior are associated with autistic traits, empathy, and Asperger syndrome. Autism Res. 2009;2(3):157–77. Epub 2009/07/15. 10.1002/aur.80 . 19598235

[91] Uzefovsky F, Shalev I, Israel S, Knafo A, Ebstein RP. Vasopressin selectively impairs emotion recognition in men. Psychoneuroendocrinology. 2012;37(4):576–80. 10.1016/j.psyneuen.2011.07.018 . 21856082

[92] van Honk J, Schutter DJ, Bos PA, Kruijt AW, Lentjes EG, Baron-Cohen S. Testosterone administration impairs cognitive empathy in women depending on second-to-fourth digit ratio. Proceedings of the National Academy of Sciences of the United States of America. 2011;108(8):3448–52. 10.1073/pnas.1011891108 21300863 PMC3044405

[93] Hysek CM, Domes G, Liechti ME. MDMA enhances "mind reading" of positive emotions and impairs "mind reading" of negative emotions. Psychopharmacology. 2012;222(2):293–302. 10.1007/s00213-012-2645-9 . 22277989

[94] Guastella AJ, Einfeld SL, Gray KM, Rinehart NJ, Tonge BJ, Lambert TJ, et al Intranasal oxytocin improves emotion recognition for youth with autism spectrum disorders. Biol Psychiatry. 2010;67(7):692–4. Epub 2009/11/10. doi: S0006-3223(09)01122-6 [pii] 10.1016/j.biopsych.2009.09.020 . 19897177

[95] Dehning S, Reiss E, Krause D, Gasperi S, Meyer S, Dargel S, et al Empathy in high-tech and high-touch medicine. Patient Educ Couns. 2014;95(2):259–64. 10.1016/j.pec.2014.01.013 . 24589130

[96] Cacciotti-Saija C, Langdon R, Ward PB, Hickie IB, Scott EM, Naismith SL, et al A double-blind randomized controlled trial of oxytocin nasal spray and social cognition training for young people with early psychosis. Schizophr Bull. 2015;41(2):483–93. 10.1093/schbul/sbu094 24962607 PMC4332939

[97] Riem MM, Bakermans-Kranenburg MJ, Voorthuis A, van IMH. Oxytocin effects on mind-reading are moderated by experiences of maternal love withdrawal: an fMRI study. Progress in neuro-psychopharmacology & biological psychiatry. 2014;51:105–12. 10.1016/j.pnpbp.2014.01.014 . 24486563

[98] Sandvik AM, Hansen AL, Johnsen BH, Laberg JC. Psychopathy and the ability to read the "language of the eyes": divergence in the psychopathy construct. Scandinavian journal of psychology. 2014;55(6):585–92. 10.1111/sjop.12138 24954681 PMC4282377

[99] Hezel DM, McNally RJ. Theory of mind impairments in social anxiety disorder. Behavior therapy. 2014;45(4):530–40. 10.1016/j.beth.2014.02.010 . 24912465

[100] Lugnegard T, Unenge Hallerback M, Hjarthag F, Gillberg C. Social cognition impairments in Asperger syndrome and schizophrenia. Schizophr Res. 2013;143(2–3):277–84. Epub 2012/12/26. 10.1016/j.schres.2012.12.001 S0920-9964(12)00662-7 [pii]. . 23266067

[101] Frick C, Lang S, Kotchoubey B, Sieswerda S, Dinu-Biringer R, Berger M, et al Hypersensitivity in borderline personality disorder during mindreading. PLoS One. 2012;7(8):e41650 10.1371/journal.pone.0041650 22870240 PMC3411703

[102] Fertuck EA, Jekal A, Song I, Wyman B, Morris MC, Wilson ST, et al Enhanced 'Reading the Mind in the Eyes' in borderline personality disorder compared to healthy controls. Psychol Med. 2009;39(12):1979–88. 10.1017/S003329170900600X 19460187 PMC3427787

[103] Koizumi M, Takagishi H. The relationship between child maltreatment and emotion recognition. PLoS One. 2014;9(1):e86093 10.1371/journal.pone.0086093 24465891 PMC3896451

[104] Harrison A, Sullivan S, Tchanturia K, Treasure J. Emotion recognition and regulation in anorexia nervosa. Clinical psychology & psychotherapy. 2009;16(4):348–56. 10.1002/cpp.628 . 19517577

[105] Oldershaw A, Treasure J, Hambrook D, Tchanturia K, Schmidt U. Is anorexia nervosa a version of autism spectrum disorders? Eur Eat Disord Rev. 2011;19(6):462–74. Epub 2011/02/01. 10.1002/erv.1069 . 21280165

[106] Russell TA, Schmidt U, Doherty L, Young V, Tchanturia K. Aspects of social cognition in anorexia nervosa: affective and cognitive theory of mind. Psychiatry research. 2009;168(3):181–5. 10.1016/j.psychres.2008.10.028 . 19467562

[107] Baron-Cohen S, Knickmeyer RC, Belmonte MK. Sex differences in the brain: implications for explaining autism. Science. 2005;310(5749):819–23. . 16272115 10.1126/science.1115455

[108] Lai MC, Lombardo MV, Chakrabarti B, Baron-Cohen S. Subgrouping the Autism "Spectrum": Reflections on DSM-5. PLoS Biol. 2013;11(4):e1001544 Epub 2013/05/01. 10.1371/journal.pbio.1001544 23630456 PMC3635864

[109] Frith U. Why we need cognitive explanations of autism. Q J Exp Psychol (Hove). 2012;65(11):2073–92. Epub 2012/08/22. 10.1080/17470218.2012.697178 . 22906000

[110] Peterson E, Miller SF. The Eyes Test as a measure of individual differences: How much of the variance reflects verbal IQ? Frontiers in psychology. 2012;3:220 Epub 2012/07/12. 10.3389/fpsyg.2012.00220 22783217 PMC3389807

[111] Baker CA, Peterson E, Pulos S, Kirkland RA. Eyes and IQ: A meta-analysis of the relationship between intelligence and “Reading the Mind in the Eyes”. Intelligence. 2014;44:78–92. 10.1016/j.intell.2014.03.001

[112] Adams RB Jr., Rule NO, Franklin RG Jr., Wang E, Stevenson MT, Yoshikawa S, et al Cross-cultural reading the mind in the eyes: an fMRI investigation. J Cogn Neurosci. 2010;22(1):97–108. 10.1162/jocn.2009.21187 . 19199419

